# Fabrication of cobalt-nickel binary nanowires in a highly ordered alumina template via AC electrodeposition

**DOI:** 10.1186/1556-276X-8-352

**Published:** 2013-08-14

**Authors:** Ghafar Ali, Muhammad Maqbool

**Affiliations:** 1 Department of Nuclear and Quantum Engineering (NQe), Korea Advanced Institute of Science and Technology (KAIST), 373-1 Guseong, Daejeon, Yuseong 305-701, Republic of Korea; 2 Department of Physics & Astronomy, Ball State University, Muncie, IN 47304, USA; 3 Nanomaterial Research Group (NRG), Physics Division, PINSTECH, Islamabad, Pakistan

**Keywords:** Electrochemistry, Anodization, Template, Electrodeposition, Magnetic, Nanowires

## Abstract

Cobalt-nickel (Co-Ni) binary alloy nanowires of different compositions were co-deposited in the nanopores of highly ordered anodic aluminum oxide (AAO) templates from a single sulfate bath using alternating current (AC) electrodeposition. AC electrodeposition was accomplished without modifying or removing the barrier layer. Field emission scanning electron microscope was used to study the morphology of templates and alloy nanowires. Energy-dispersive X-ray analysis confirmed the deposition of Co-Ni alloy nanowires in the AAO templates. Average diameter of the alloy nanowires was approximately 40 nm which is equal to the diameter of nanopore. X-ray diffraction analysis showed that the alloy nanowires consisted of both hexagonal close-packed and face-centered cubic phases. Magnetic measurements showed that the easy *x*-axis of magnetization is parallel to the nanowires with coercivity of approximately 706 Oe. AC electrodeposition is very simple, fast, and is useful for the homogenous deposition of various secondary nanostuctured materials into the nanopores of AAO.

## Background

Porous anodic aluminum oxide (AAO) attracted a remarkable interest due to the pioneer work of Masuda and Fukuda [1]. Self-organized nanoporous structure with hexagonal ordered morphology can be obtained on a highly pure Al surface via electrochemical anodization in acidic medium [1,2]. AAO is extensively applied in the fields of biosensor [3] and biofiltration [4] and as a nanotemplate [5,6] for the fabrication of secondary nanostructured materials. AAO templates have many advantages over the polycarbonate membranes like high pore density, thermal stability, cost effectiveness and versatility. Pore diameter, length, inter-pore spacing, and pore ordering can be easily tailored by tuning the anodizing parameters such as voltage, time, electrolytes, pH value, and temperature.

One-dimensional (1D) nanostructured materials such as nanowires, nanorods, and nanotubes play a special role in the field of nanoscience and nanotechnology due to their high aspect ratio (length/diameter) and large surface area. Ferromagnetic (Fe, Co, Ni) nanowires gain a lot of attention of scientific community in the last few decades due to their potential application in the fields of ultra-high density magnetic storage [7], magnetio-electronics [8], high sensitive giant magnetoresistance (GMR) sensors [9,10]. Co–Ni is an important type of binary ferromagnetic alloys having high mechanical strength [11], good wear resistance [12], anti-corrosive performance [13], and electrocatalytic activity [14,15]. Moreover, the standard electrochemical potentials of Co^2+^ and Ni^2+^ almost have the same value of −0.28 and −0.23 V, respectively, so Co–Ni binary alloy nanowires can be easily fabricated in the nanopores of AAO template by co-electrodeposition. Information technology made much progress especially in the last few years, which reflects the interest of the researchers and investment of companies in this field. A decade ago, the limit of areal density was about few 10 gigabits (GB)/in.^2^[16]. Today, the limit reached to several hundred GB/in.^2^. Terabit (TB) hard disk is already available commercially, and a number of companies are in competition to increase the capacity and decrease the size of the hard disk [17]. The areal density has been increased using nanomagnet, in which 1 bit of information corresponds to a single-domain nanosized particle. One simple and economical way of achieving nanomagnetic arrays over a large area is based on highly ordered AAO templates [16]. Up till now, several methods have been applied to fill the pores of AAO template with metallic or magnetic nanowires like sol–gel [18], chemical vapor deposition [19], electroless deposition [20], and electrochemical deposition [5]. Electrochemical deposition is the most simple, efficient, versatile, and cost effective technique. It is well known that anodization of metals is always associated with an insulting barrier layer between the metal substrate and metal oxide film [21]. Thickness of the barrier layer mainly depends upon the anodizing voltage with a growth rate of 1 nm/V [22]. It has been reported that the insulting properties of the barrier layer significantly affect the uniformity and quality of the depositing material [23]. Therefore, handling of the barrier layer during deposition of secondary material in the nanopores of AAO is very essential and important. Until now, three different kinds of electrochemical deposition methods are applied for filling the pores of AAO template: direct current (DC) electrodeposition [24], pulse electrodeposition (PED) [25], and alternating current (AC) electrodeposition [26]. Filling of AAO pores with metallic or magnetic nanowires via direct current (DC) electrodeposition is a tedious process and requires many steps. For instance, first AAO template has to be isolated from Al substrate, and this is achieved by dissolving the Al substrate in a toxic saturated solution of HgCl_2_. Subsequently, the barrier layer has to be etched away using chemical etching which often leads to the non-uniform widening of pores at the bottom. This process produces AAO template with different pore diameters at the top and the bottom surface; resulting in non-uniform-diameter nanowires which is undesirable in device fabrication. Finally, a thin metallic contact is sputtered on one side of AAO which act as a cathode during electrodeposition. These steps are time consuming, and additionally, the handling of a fragile AAO template during the whole process is a very difficult task. Furthermore, electrodeposition via direct current in the pores of AAO without modification of barrier layer is generally unstable and leads to a non-uniform filling of the AAO nanopores due to the cathodic side reaction [25]. PED method is also widely used for the fabrication of metallic or magnetic nanowires in the nanopores of AAO templates. Ni [16,25] and Co [27,28] nanowires have been fabricated in the nanopores of AAO applying this method. Although the uniformity and pore-filling efficiency increased many folds compared to DC electrodeposition; however this method also needs modification of the barrier layer [16,25-28]. In contrast, AC electrodeposition is a very powerful technique and it does not need the detachment of AAO template from the Al-substrate or modification of the barrier layer. Moreover, the Al-substrate is used as cathode during electrodeposition.

To the best of the author knowledge, Co-Ni binary alloy nanowire electrodeposition in the AAO template without modification of the barrier layer has not been reported to date. In this study, the fabrication of dense Co-Ni binary alloy nanowires within the nanopores of AAO templates via AC electrodeposition has been reported. Co-Ni binary alloy nanowires with different composition were co-deposited into the nanopores of AAO templates from a single sulfate bath of Co and Ni without modifying the barrier layer at room temperature.

## Methods

### Materials

Aluminum foils (Goodfellow, 0.1-mm thickness, 99.999% purity), sulfuric acid (H_2_SO_4_, Sigma-Aldrich, 99.999%, St. Louis, MO, USA), cobalt (II) sulfate heptahydrate (CoSO_4_·7H_2_O, Sigma-Aldrich, ≥99%), nickel (II) sulfate hexahydrate (NiSO_4_·6H_2_O, Sigma-Aldrich, 99%), boric acid (H_3_BO_3,_ Sigma-Aldrich, ≥99.5%) were used in their as-received forms without further treatment. The electrolyte was prepared with deionized (DI) water.

### Preparation of AAO templates

For all experiments, Al foils were cut into 4.5 × 4.5 cm^2^ pieces. Before anodization, Al foils were annealed at 500°C for 5 h in air to remove the mechanical stresses. Subsequently, the foils were etched in 1.0 M NaOH at room temperature until bubbles over the surface of the foils were observed, followed by a rinse in DI water many times and dried by air at high pressure. Al foils were used for anodization without any pre-treatment of electro-polishing. A simple, homemade, two-electrode system, with Al foil as a working electrode and a Pt foil as a counter electrode, was used for an electrochemical anodization. A circular shape surface of the Al foil was exposed to the electrolyte. Anodization was conducted in 0.4 M aqueous H_2_SO_4_ electrolyte at constant voltage of 26 V for 23 h using a DC power source at 0°C. The anodization induced highly ordered nanopores with hexagonal morphology over the exposed surface of Al foil to the electrolyte. The templates were washed with DI water and dried using air at high pressure before deposition of Co-Ni binary alloy nanowires.

### Deposition of Co-Ni binary nanowires

Co-Ni binary alloy nanowires were co-deposited in the nanopores of AAO by AC electrodeposition using a homemade, two-electrode system. In order to fabricate Co-Ni alloy nanowires in the nanopores of AAO templates, a single sulfate bath containing 50 mL of aqueous solution (mixture) of CoSO_4_·7H_2_O and NiSO_4_·6H_2_O was used as a source of cobalt and nickel ions. For the fabrication of Co-Ni binary nanowires of different composition, the concentration ratios of Co(II) to Ni(II) was varied in the reaction solutions as given in the Table 1. A small amount of H_3_BO_3_ (1.5 g/L) was added in each solution bath to prevent hydroxide formation and facilitate the deposition procedure. During the co-deposition process, the open side of AAO templates was placed in contact with the electrolyte solution. A graphite disc was used as a counter electrode and AAO templates with remaining aluminum at the back as a working electrode. Before electrodeposition, the solutions were constantly stirred for a few minutes. Electrodeposition in the AAO templates was carried out at room temperature using AC voltage of 15 V_rms_ for 5 to 10 min with current density of 15 mA at 50 Hz. The co-electrodeposition process filled the nanopores of AAO templates with Co-Ni materials. The AAO templates containing Co-Ni binary nanowires were washed with DI water and dried. Finally the AAO templates were dissolved with the help of NaOH.

**Table 1:** Concentration ratio of Co(II) to Ni(II) in the bath solution

**Solution No.**	**Concentration ratio of the metallic species in the bath**	**Amount of powder in the reaction solution (g/L)**	**Final solution (mL)**
	**[Co(II)/Ni(II)]**	**Co/Ni**	
1	90:10	12.6:1.3	50
2	80:20	11.2:2.6	50
3	70:30	9.8:3.9	50
4	60:40	5.2:8.4	50
5	50:50	6.5:7.0	50

### Characterization

The structural morphology of AAO templates and Co-Ni binary nanowires was studied with the help of field emission scanning electron microscope (FESEM, Magellan, FEI, USA). The cross-sectional SEM images were taken from mechanically cracked samples. Elemental analysis was done using an energy dispersive X-ray analyzer (EDX) analyzer attached onto the SEM. The crystallographic structure of the nanowires were determined by a high-power X-ray generator (18 kW) Rigaku D/MAX-2500 X-ray diffractometer (Shibuya-ku, Japan) with Cu Kα radiation (*λ* = 1.54056 Å). The magnetic properties were measured with the help of vibrating sample magnetometer (Lake Shore 7407, Westerville, OH, USA) at room temperature.

## Results and discussions

**Figure 1:**
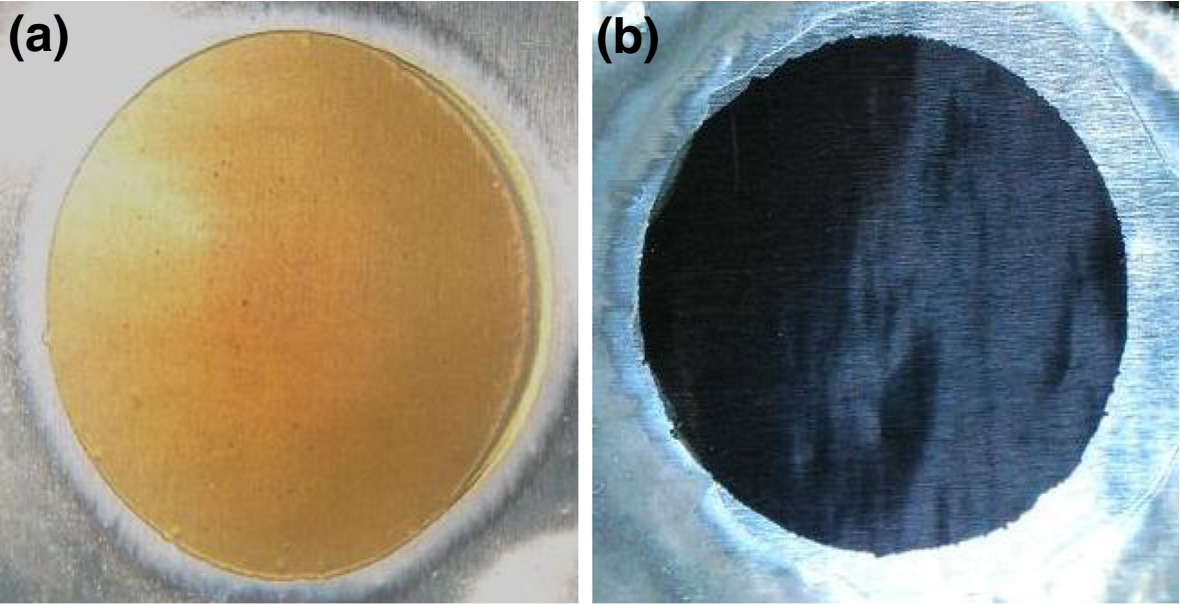
Digital photos of AAO template without (a) and with (b) Co-Ni binary nanowire co-deposition.

**Figure 2:**
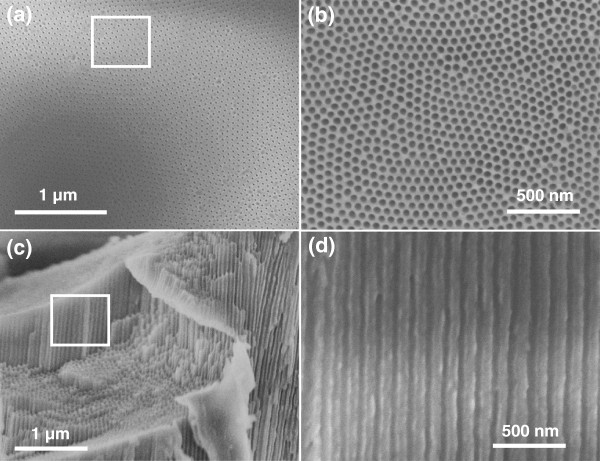
**FESEM image of AAO template. (a**,**b)** Top surface view at low and high magnification. **(c**,**d)** Cross-sectional view at low and high magnification.

**Figure 3:**
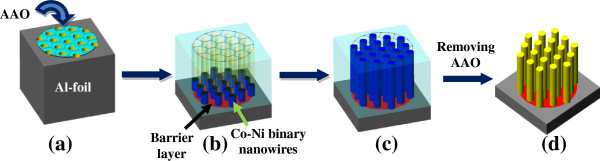
**Schematic diagram for co-deposition process of Co-Ni binary nanowires in nanopores of AAO template. (a)** AAO template with circular shape, **(b)** filling of nanopores started from Co-Ni binary nanowires at the bottom of AAO by exposing circular area to the Co and Ni precursor solution, **(c)** complete filling of the alumina nanopores from Co-Ni binary nanowires, **(d)** dissolution of alumina in NaOH to get Co-Ni binary nanowires.

Figure 1 shows digital photos of the AAO template before (Figure 1a) and after Co-Ni metallic deposition (Figure 1b) using alternating current. It shows that template has completely gone black after electrodeposition, confirming the metallic deposition. Figure 2 shows SEM micrographs of the top and cross sectional surfaces of AAO template at different magnifications. Low magnification top surface image (Figure 2a) shows that the nanopores are very dense and uniform with perfect hexagonal ordering. High-magnification image of the top surface (Figure 2b) clearly exhibits the pore ordering and their geometry. All the pores are in circular shape with average pore diameter of approximately 40 nm and average inter-pore distance of approximately 65 nm. Figure 2c,d shows the cross-sectional images of AAO template which reveals that the nanopores or nanochannels are very straight and parallel throughout their entire length. The width of nanochannel (Figure 2d) corresponds to the diameter of the nanopore in the top surface view image (Figure 2b). Figure 3 gives a schematic diagram of the metallic deposition process in a highly ordered AAO template (Figure 3a) via AC deposition process. The main advantage of this method is to avoid the complex process of Al and barrier layer removal prior to deposition as described earlier in the introduction section. The nanopores of AAO started filling from the bottom with Co-Ni materials when the AC voltage power supply is switched on (Figure 3b). Metal precursors of Co that is Co^2+^ and Ni (Ni^2+^) were diffused from the single sulfate solution in the nanopores of AAO with the help of an applied electric field (AC voltage). These metal precursors reduced to Co and Ni at the Al surface via the following chemical reactions:

(1) $${\mathrm{Co}}^{2+}+{2e}^{‒}\;\to \;\mathrm{Co}$$

(2) $${\mathrm{Ni}}^{2+}+{2e}^{‒}\;\to \;\mathrm{Ni}$$

Metallic cobalt and nickel give an intermetallic phase according to the following reaction [29]:

(3) Co+Ni→CoNi

**Figure 4:**
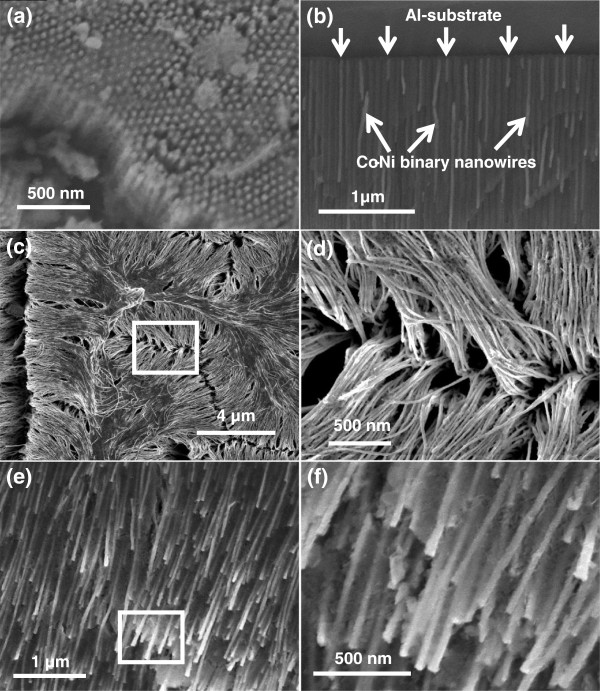
**SEM images of Co-Ni binary nanowires. (a)** top surface and **(b)** cross-sectional view embedded in AAO template, **(c**, **d)** top surface view of Co-Ni binary nanowires partially liberated from AAO template at low and high magnification **(e**, **f)** tilted view at low and high magnifications.

**Figure 5:**
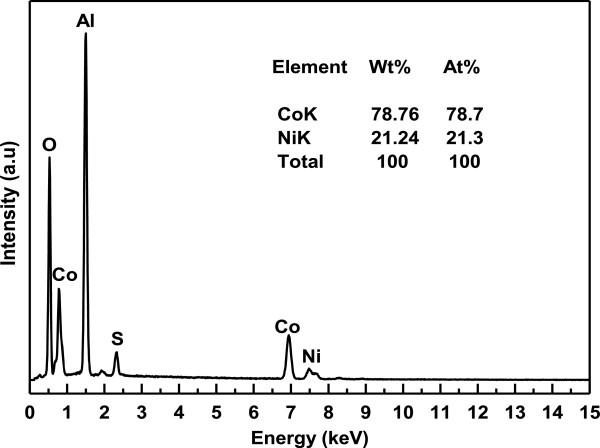
**EDX spectrum of Co-Ni binary nanowires [Co(II)/Ni(II) = 80:20].** Embedded in AAO template along with their quantitative analysis.

**Figure 6:**
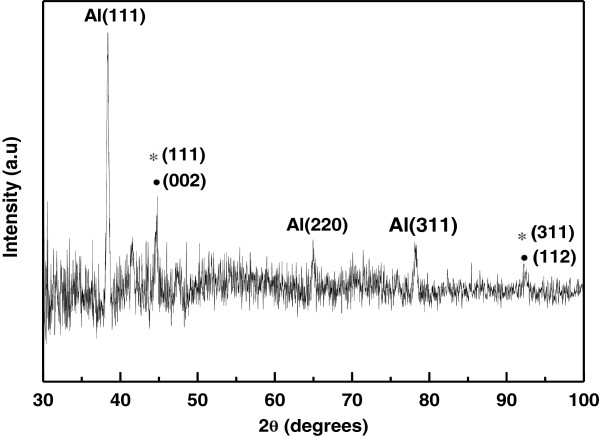
**XRD pattern of the Co-Ni binary nanowires embedded in AAO template.** Asterisks indicate fcc, while solid black circles indicate hcp Co-Ni binary nanowires.

**Figure 7:**
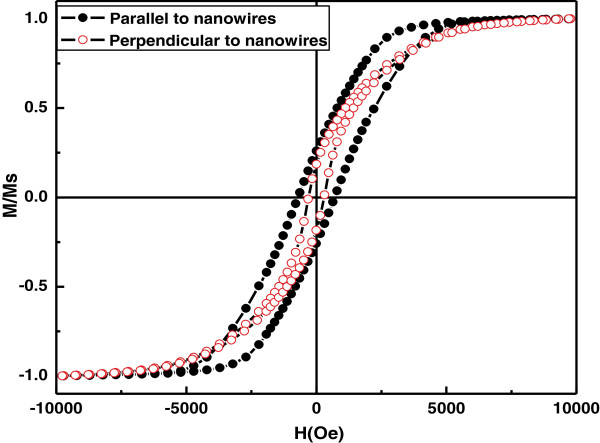
**Hysteresis loops of Co-Ni binary nanowire [Co(II)/Ni(II) = 80:20].** Measured at room temperature using vibrating sample magnetometer.

It is important to mention that deposition of metal precursors started in the nanopores of AAO only when the polarity of the electrodes is reversed unlike anodization. The electrodeposition process was continued until the nanopores are filled completely with Co-Ni materials (Figure 3c). It is worth noticing that the deposition time must be controlled to suppress the outer grow of depositing material from the AAO template and subsequent cap formation [30,31]. Such bottom-up growth process fills all the nanochannels of AAO with Co-Ni material, resulting in the formation of Co-Ni binary nanowires (Figure 4). Finally Co-Ni binary nanowires were liberated by dissolving the AAO template (Figure 3d). The morphology of Co-Ni binary nanowires is shown in Figure 4. Figure 4a shows SEM image of the top surface of Co-Ni binary nanowires embedded in AAO template. It can be seen from the image that the nanopores of AAO template are filled completely with Co-Ni binary nanowires showing the uniform deposition and homogeneity of the nanowires by AC electrodepsoition. It clearly shows that the growth of Co-Ni binary nanowires was restricted into the nanopores of AAO and suppressed the subsequent cape formation at the top. Figure 4b shows the cross-sectional image of Co-Ni binary nanowires embedded in the alumina template giving a bright contrast as marked by arrows. Few nanochannels without Co-Ni binary nanowires can also be seen in the image. This indicates that some Co-Ni binary nanowires have been broken and removed from the AAO template. Breaking and removal of Co-Ni binary nanowires from the alumina nanochannels is attributed to the mechanical stress applied during the preparation of sample for cross-sectional view in SEM. Since the sample was simply cut with scissor, the empty alumina nanochannels might indicate that Co-Ni binary nanowires were embedded in the other half portion of the alumina template. Moreover, the image verifies that the deposition of Co-Ni binary nanowires start from the bottom surface of alumina nanochannels as explained in the Figure 3b. The marked area near the Al substrates (Figure 4b) represents the bottom part of the Co-Ni binary nanowires which confirm the deposition without the modification of the barrier layer. Figure 4c,d shows the top surface view of Co-Ni binary nanowires after partial dissolution of AAO template. It can be seen from the images that the nanowires are very copious and ultra-long, and their density corresponds to the density of the nanopores (Figure 2a). The diameter of the nanowires is relatively uniform along their entire length and equal to the diameter of alumina nanopores (approximately 40 nm). Figure 4e,f represents the tilted images of Co-Ni binary nanowires partially separated from the AAO template. It further verifies the suppression of cape formation over the top surface of Co-Ni binary nanowires. These results show that the most of the nanochannels of alumina are successfully filled with Co-Ni binary nanowires and have continuous morphology without any intermittence contrary to the chain-like CoNi alloy wires [29,32,33]. The formation of Co-Ni alloy nanowires has been confirmed using EDX. EDX analysis of Co-Ni binary nanowires [Co(II)/Ni(II) = 80:20] embedded in the AAO template is given in Figure 5. The characteristic peaks in the spectrum are associated with Co, Ni, Al, O, and S. Co and Ni peaks arise from the co-deposited Co-Ni binary nanowires, while O and Al peaks are appearing from the matrix of alumina template, and S peak is due to the use of sulfuric acid as electrolyte for anodization. The quantitative analysis obtained from EDX analysis is almost close to the concentration ratio of the metallic species in the reaction solution. Figure 6 shows the X-ray diffraction (XRD) pattern of Co-Ni binary nanowires embedded in the AAO template for [Co(II)/Ni(II) = 80:20] system. Both hexagonal close-packed (hcp) and face-centered cubic (fcc) peaks observed in the XRD pattern (JCPDS 05–0727 and 04–0850). Generally, cobalt is stabilized in the hcp structure at room temperature. Kawamori et al. [32] found both hcp and fcc phases in the Co-Ni alloy nanoparticles and nanowires prepared using electroless disposition under magnetic field. They further reported that both hcp and fcc phases are the equilibrium phase at Co/Ni = 70:30 (atom%) which is close to our system composition. This result has been further verified from the binary phase diagram of Co-Ni. A mixed structure of hcp and fcc phases has been observed in the binary phase diagram of Co-Ni at Co_71_Ni_29_ alloy composition. Interestingly, peaks corresponding to pure Co and Ni have not been observed in the XRD pattern which shows that Co and Ni formed an alloy instead of existing in separate grains. The background noise observed in the XRD pattern originates from the amorphous nature of AAO [34]. Figure 7 shows the typical hysteresis loop of Co-Ni binary nanowires [Co(II)/Ni(II) = 80:20] embedded in the AAO template measured at room temperature at magnetic field of ±10 kOe applied both parallel and perpendicular to the nanowire axis. It can be seen from the figure that the square shape of the loop and widening is more in case when the field was applied parallel to the wire axis compared to the perpendicular direction. This shows that the easy axis of magnetization is along the nanowires while hard axis is normal to the nanowires. Coercivity (H_C_) values of the nanowires in parallel and perpendicular direction are approximately 706 and 298 Oe, respectively, which are higher than the reported value of Co-Ni alloy wire [29,32] and Co-Ni powders [35]. The higher value of H_C_ in case of easy axis is attributed to the fact that in such case the domains are lying along the axis of the nanowires. This favors the easier alignment (and reversal) of magnetic spins along the applied field direction causing a broad and squared hysteresis loops. It is worthy to note that the crystalline anisotropy (as well as the shape anisotropy) reinforces each other and both seem to align along the easy axis of the nanowires. The square shape and widening of the MH-loop of Co-Ni binary nanowires is smaller than the pure Co-nanowires [5]. This is attributed to the strong magnetic interactions among the Co-nanoparticles comprising the Co-nanowires [5,32] compared to Co-Ni nanoparticles comprising the Co-Ni binary nanowires. These nanowires will also be used in the future to produce nanolaser after depositing lasing materials on them. Maqbool has already reported titanium-doped infrared microlaser on optical fibers [36]. Using the same idea this time, we will use these Co-Ni nanowires to produce nanolaser.

## Conclusion

In summary, dense Co-Ni binary alloy nanowires were deposited into highly hexagonal ordered nanopores of AAO template via AC electrodeposition at room temperature without barrier layer modification. Hexagonal ordered AAO templates were synthesized in 0.4 M H_2_SO_4_ at 26 V in 0°C environment via single-step anodization. Co-Ni binary alloy nanowires were homogenously co-deposited within the nanopres of AAO template from a single sulfate bath. FESEM results showed that the nanowires have uniform lengths and diameters. Diameters of the nanowires were approximately 40 nm which is equal to the nanopore diameter. XRD analysis confirmed the fabrication of Co-Ni binary alloy nanowires with hcp and fcc phases. EDX analysis confirms the fabrication of Co-Ni binary alloy nanowires in the AAO template. Magnetic measurement showed that easy *x*-axis of magnetization is along the parallel direction of the nanowires with coercivity of approximately 706 Oe. The AC deposition method is very simple, fast, and cost effective as it does not need the complex process of barrier layer removal or modification for the deposition of secondary nanostructured materials in the nanopres of AAO.

## Competing interests

The authors declare that they have no competing interests.

## Authors’ contributions

GA carried out the experiments, participated in the sequence alignment and drafted the manuscript. MM conceived of the study and participated in its design and coordination. Both authors read and approved the final manuscript.
